# Food Supply without Risk: Multicriteria Analysis of Institutional Conditions of Exporters

**DOI:** 10.3390/ijerph17103432

**Published:** 2020-05-14

**Authors:** Rosa Puertas, Luisa Marti, Jose-Maria Garcia-Alvarez-Coque

**Affiliations:** Group of International Economics and Development, Univèrsitat Pòlitecnica de València, Camí de Vera, s/n, 46022 València, Spain; mlmarti@esp.upv.es (L.M.); jmgarcia@upvnet.upv.es (J.-M.G.-A.-C.)

**Keywords:** food safety, trade, supply chain, multicriteria decision analysis

## Abstract

International trade in food knows no borders, hence the need for prevention systems to avoid the consumption of products that are harmful to health. This paper proposes the use of multicriteria risk prevention tools that consider the socioeconomic and institutional conditions of food exporters. We propose the use of three decision-making methods—Technique for Order Preference by Similarity to the Ideal Solution (TOPSIS), *Elimination et Choix Traduisant la Realité* (ELECTRE), and Cross-Efficiency (CE)—to establish a ranking of countries that export cereals to the European Union, based on structural criteria related to the detection of potential associated risks (notifications, food quality, corruption, environmental sustainability in agriculture, and logistics). In addition, the analysis examines whether the wealth and institutional capacity of supplier countries influence their position in the ranking. The research was carried out biannually over the period from 2012–2016, allowing an assessment to be made of the possible stability of the markets. The results reveal that suppliers’ rankings based exclusively on aspects related to food risk differ from importers’ actual choices determined by micro/macroeconomic features (price, production volume, and economic growth). The rankings obtained by the three proposed methods are not the same, but present certain similarities, with the ability to discern countries according to their level of food risk. The proposed methodology can be applied to support sourcing strategies. In the future, food safety considerations could have increased influence in importing decisions, which would involve further difficulties for low-income countries.

## 1. Introduction

The internationalization of the food trade has evolved in parallel with concerns about quality, prompting the development of techniques to assess and prevent the risk associated with the transmission of pathogens and address other safety issues. Agencies such as the Codex Alimentarius Commission (CAC), the United Nations Food and Agriculture Organization (FAO), and the World Health Organization (WHO), among others, have been collaborating for more than three decades in an effort to protect health and ensure good practices in the trade of food products [1]. They all recommend taking a preventive approach involving hazard analysis and critical control points (HACCP) as a precautionary system and a means of guaranteeing food safety [2,3]. Rohr, J.R., et al., underline that feeding the human population will require paying more attention to food-related research, management, and policy to assure human health [4]. De Jonge, J., et al., emphasize that consumer confidence in food safety heavily depends on trust in institutions and organizations [5]. The perceived risk in supplying countries is a determining factor in food purchases [6]. To assess food risk, it is necessary to broaden the focus beyond the traditional control in internal manufacturing processes, toward actions aimed at prevention and reducing vulnerability. The objective of this paper is to provide food importers with a multicriteria framework for risk assessment and prevention based on institutional and economic indicators of product suppliers. Trade in agricultural products entails long distribution chains, where so-called food miles—the distance food has to travel between producer and consumer—are rising at a rate that calls for adaptation by all actors involved [7]. Therefore, the choice of the supplier requires an analysis of multiple, widely differing factors, on a potentially huge scale. Moreover, making the wrong choice could lead to major economic and social disruption for both parties. 

The justification for this research is that there is an emerging need for a systematized approach to carrying out food risk assessment that takes into account the economic and policy conditions of exporters. Wood, V.R., et al., identified 200 indicators that influence the choice of the supplier country, making it possible to distinguish international target markets [8]. The FAO has already produced guidance materials for decision-makers regarding the most dangerous factors, establishing a structured process for detection and the adoption of strategies to ensure food quality [9]. Jouanjean, M.A., et al., refer to the “reputation effects” to explain how the history of food controls or notifications by food importing authorities at borders can predict future notifications [10]. A risk assessment can include a range of local conditions such as those related to the level of corruption in the supplier country, the historical evidence of noncompliance, and the existence of control policies [11]. None of the quoted sources include multicriteria decision-making (MCDM) to be used by importing firms facing supplier selection decisions.

As far as international trade is concerned, some econometric studies have introduced policy and institutional variables for forecasting food incidents and alerts [12,13,14,15,16]. However, while most of these approaches are helpful in providing an ex post explanation of the drivers of food risk, their focus has not been the ex ante multicriteria assessment of risk drivers for prevention purposes. 

In this paper, a multicriteria analysis tool is proposed to assist panels of experts of importing firms to select product suppliers according to their domestic institutional conditions. The framework is able to rank supplying countries according to different domestic markers based on their logistical complexity, state of food quality, level of development, environmental conditions, and reputation in terms of the history of previous safety notifications for their exports of a given food product. Our purpose is to provide a tool for risk prevention that improves the exchange of information between all stakeholders, advisors, managers, and even consumers [17,18,19,20].

This research paper proposes the use of three MCDM methods—Technique for Order Preference by Similarity to the Ideal Solution (TOPSIS), *Elimination et Choix Traduisant la Realité* (ELECTRE), and cross-efficiency (CE)—to establish a ranking of non-European Union countries supplying cereals to the European Union in 2012, 2014, and 2016. The study employs innovative discrimination criteria: instead of using micro/macroeconomic parameters to produce a ranking based on business trends, the applied criteria characterize the quality of the product and the potential food risk associated with the exporting country. In this way, it is possible to indicate suppliers whose products pose the least danger to consumer health. This research expands the current paradigm surrounding food risk, in which MCDM methods have been mainly applied to topics related to food safety, policies designed to improve microbial food safety, and identifying obstacles to food safety policies [21,22,23].

The use of multicriteria models to choose suppliers in the food sector has been studied in the literature [24,25,26,27]. However, no research to date has focused exclusively on the selection criteria for securing a food supply without risk, avoiding purely business issues. The proposed research contribution aims to provide concrete solutions to real problems, such as choosing countries that guarantee the supply of safe products. 

We take cereals as an interesting value chain to apply the proposed framework. Cereals are products of paramount relevance to human consumption, animal feed, and pet food. As with any food that can be processed for consumption, there is a need to ensure quality and safety throughout the supply chain. It is a product of great relevance in European trade, as shown by official United Nations statistics through Comtrade. Specifically, the value of cereal imports by the EU ($6523 million in 2016) exceeds other food imports, such as dairy products and eggs ($712 million), sugar ($2870 million), and meat ($6024 million). It is therefore a widely consumed product that a priori can seem to be without risk; nevertheless, its consumption in a bad state would seriously harm the health of the population.

In summary, the paper makes a number of contributions to the literature: (1) It provides a systematized approach to risk assessment that integrates basic institutional conditions linked to the likelihood of potential risks in the country of origin; (2) The conditions are integrated into a multicriteria decision-making process valid for food importers; (3) The framework combines three methodologies to strengthen the conclusions obtained; (4) The methodology makes it possible to rank cereal suppliers based on several criteria (number of notifications at the border, food quality, perceived level of corruption in the country of origin, environmental sustainability in agriculture, and logistics) that can be used to inform the decision-making of food importers; and (5) The application provides an analysis of how the development and institutional capacity of cereal exporters determine their position in the ranking. This approach differs from the most common food safety techniques by anticipating, through signaling country risks, the problems that may arise.

The rest of the paper is organized as follows: Section 2 provides a literature review, with a focus on multicriteria methods applied to food issues. Section 3 explains the methodology and sample used in the empirical analysis. Section 4 presents the results obtained. Finally, Section 5 summarizes the main findings of the study.

## 2. Literature Review

Over the past two decades, the EU has focused on developing and strengthening risk assessment in order to adapt guidelines to existing needs [28,29]. In particular, it has developed a set of rules to regulate the production, processing, and distribution chains of both domestic and imported food, establishing a series of controls to ensure compliance (Green Paper, General Principles of Food Law in 1997; Consumer Health and Food Safety in 1997; Food Safety Campaign in 1998; White Paper on Food Safety in 2000; General Food Law, GFL, in 2002). Furthermore, the EU has considered food security in the Common Agricultural Policy (CAP), although this concept has been widely criticized for its ambiguity [30,31,32]. As shown in the literature, ensuring food safety/security is a complex problem, and while there is consensus that the CAP should treat it as a strategic objective, there is a lack of unanimity on the most appropriate course of action or a specific way to implement it [33,34]. 

The EU has developed controls on cereals; both European-grown and imported cereals are subject to strict regulatory standards, due to their high exposure to pollutants. Notifications at borders that are reported by the member state authorities to the Rapid Alert System for Food and Feed (RASFF) go some way toward ensuring that food is imported in perfect condition. 

While institutional conditions have been considered for the quantitative forecasting of RASFF notifications for fresh food products [14], nuts [16], and the whole agri-food sector [35], little has been published on practical tools to assist food importers or policy-makers with their assessment of how the political and structural conditions of supplying countries can affect the safety of their exported products. Some guidelines have been designed to consider sociopolitical opportunities and motivations for food fraud [11,36,37]. 

Multicriteria decision-making (MCDM) methods are ideal tools for gaining a better understanding of decision-making processes, facilitating comparisons between alternatives. According to [38], the central problem lies in evaluating a set of alternatives in terms of multiple criteria. As a result, these methods have been applied in the fields of economics [39,40,41,42,43,44,45], energy fuels [46,47], environmental sciences [48], and engineering and transportation [49,50,51], as well as in universities [52,53]. Table 1 summarizes relevant contributions to the literature where multicriteria techniques have been used to support decision problems related to the agricultural and food sectors. 

In the literature, there is a lot of work aimed at establishing rankings of countries, suppliers, and/or indicators by means of MCDM methods, in order to facilitate the choices of decision-makers. However, little attention has been given to the social, political, and institutional conditions affecting food risk. Multicriteria decision-making has scarcely been used as a risk prevention tool in agricultural trade, and even less to weigh the socioeconomic and institutional conditions of the country of origin. The proposed decision-making framework in this paper is aimed at covering a less worked aspect which is of vital importance to ensuring the safety of the population, providing a tool for assessing the food risk and introducing exporter’s institutional variables into evaluations. The use of different methodologies reinforces the conclusions obtained, identifying supplier countries whose products are more likely to carry pathogens and addressing other issues that invalidate the safety of the product.

## 3. Materials and Methods

MCDM methods cover a wide variety of approaches and can be classified into two broad categories: discrete MCDM, also known as multi-attribute decision-making (MADM), and continuous MCDM, or multiobjective optimization problems (MOOPs). The latter methods are associated with problems where the alternatives are not predetermined, with the aim of designing the best option, taking into account a set of quantifiable objectives; they include goal programming, multiple objective programming, and compromise solution methods [64]. These problems can be solved using single-level, fuzzy, multistage, and dynamic methods. Discrete methods are associated with rational choice theory, by which individuals are motivated to gain some benefit, acting rationally, and are limited by a series of conditioning factors [65]. A distinction is made between structural relationship methods (AHP, DEMATEL, ANP, ISM or entropy measure) and performance aggregate methods (SAW, TOPSIS, ELECTRE, PROMETHEE, VIKOR). 

Data envelopment analysis (DEA), on the other hand, can be used to solve problems with multiple inputs/outputs that characterize a set of observations, enabling a ranking of the observations, like the aforementioned MCDM models [66]. DEA has proven to be widely applicable as a decision-making tool, and has been extensively used to solve management problems involving choices between different alternatives [67,68,69,70]. 

As [71] pointed out, these techniques are considered part of operational research. [72] carried out a comparison of TOPSIS, ELECTRE, and AHP, concluding that the first two yield similar results in problems involving the choice of a location. Along the same lines, a number of studies were aimed at comparing these techniques, but did not report conclusive results [73,74,75,76].

**Figure 1:**
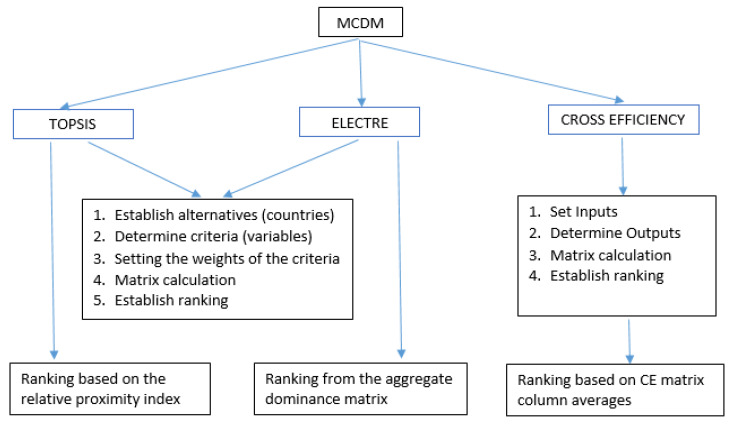
Diagram of multicriteria techniques. Source: Authors’ elaboration.

This study proposes the use of TOPSIS, ELECTRE, and CE, all of which have proven to be suitable for solving problems that require the calculation of a ranking for a set of alternatives. Furthermore, they are comparable when applied to the same sample of exporters and provide robustness to the conclusions obtained. A diagram with the stages of each method is shown in Figure 1.

It is important that the alternatives are well defined and the criteria to be evaluated are correctly set. When implementing TOPSIS and ELECTRE, weights must be assigned to the criteria, depending on their importance in the final result. DEA, on the other hand, requires the identification of inputs/outputs that define the production function, allowing a reference frontier to be identified and the subsequent ranking of observations to be carried out [77,78]. Table 2 compares the three methods in terms of descriptions, advantages, and disadvantages. The three methods are described in detail in the Appendix A.

**Figure 2:**
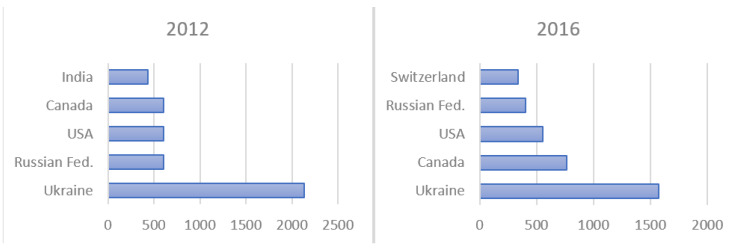
Main suppliers of cereals to the EU ($ mill). Source: Authors’ elaboration. Comtrade.

Comtrade data on the origin of imports in 2012 and 2016 indicate that Ukraine was the main European supplier of cereals, accounting for more than $2 billion worth in 2012, and slightly less in 2016 (Figure 2). Since 2011, this country has had abundant harvests and a drastic drop in its population (both factors that have helped boost its exports), while it has also received assistance from the FAO, the European Bank for Reconstruction and Development, and the EU4Business initiative. At the same time as their cereals were being promoted, Ukrainian producers were advised on issues related to state policies, technology, regulations, and standards, all of which resulted in remarkable growth in their international sales [84].

The main EU suppliers remain fairly stable, with Canada, the USA, Russia, and Ukraine dominating in both 2012 and 2016 (Figure 1). The EU has free trade agreements (FTAs) with some countries, thereby strengthening trade relations. FTAs open up markets for agricultural products, foods, and beverages, thus providing value and creating jobs in both the primary agriculture and food processing sectors [85]. Based on the data provided by the statistics, it bears asking whether EU suppliers are suitable for the EU in terms of a number of criteria other than price policy and certain micro/macroeconomic issues, namely, issues more closely related to food risk. MCDM was selected due to its advantages in decision-making processes that involve different criteria for the choice of the most appropriate alternative. In short, as stated in the Introduction, this approach produces a ranking of countries that facilitates the choice of the most suitable cereal supplier in terms of food risk. 

The empirical analysis focused on a sample of countries that constituted the main non-EU suppliers of cereals to the EU in 2012, 2014, and 2016. It was constructed using information on cereal imports provided by Comtrade (United Nations). Table 3 presents the ranking of countries according to the volume of cereals exported (from highest to lowest), according to the micro/macroeconomic criteria of official statistics.

Following the sample construction process, for each year under study, 30 suppliers were initially selected, representing around 97% of EU cereal imports. However, due to the criteria used for the ranking, the sample had to be reduced to 24, 22, and 25 observations for 2012, 2014, and 2016, respectively. The set of suppliers constitutes alternatives for the models used, while the criteria were determined by the following economic, environmental, and institutional conditions related to their level of food risk (Table 4).

The analysis of notifications was carried out using information published in the RASFF database. This is a platform that registers all products imported by European countries that either do not comply with EU regulations or simply offer reasonable grounds upon which to suspect a lack of compliance. The system is key to ensuring the cross-border monitoring of goods, and enables rapid reaction when public health risks due to alterations in food or feed consignments are detected in the food chain. The number of notifications reveals the perceived historical records by EU border controls on cereal suppliers’ safety. The information was extracted from the RASFF database, with the search being constrained to the years considered in the analysis, cereals as the only type of product, and the receipt of any type of safety notification.

The Logistics Performance Index (LPI), published by the World Bank, provides information on the logistics of 160 countries [86,87,88]. The index values range between 0 and 5; a country with the highest possible level of logistics development would score 5. The score is the result of a qualitative assessment by a group of experts analyzing the following components: customs, infrastructure, international shipments, logistics quality and competence, tracking and tracing, and timeliness. The LPI values correspond to the indices published in 2014, 2016, and 2018, since they refer to the years of the present study.

The quality and safety index (Q&S) is one of the four pillars that make up the Global Food Security Index published by the Economist Intelligence Unit; the other three dimensions are affordability, availability, and natural resources and resilience. It comprehensively examines 113 countries, establishing a ranking based on 26 indicators. This study uses the Q&S dimension corresponding to the 2012–2016 period, which, in addition to nutritional issues, assesses aspects related to food safety.

The Corruption Perceptions Index (CPI) classifies countries according to their perceived level of public sector corruption, as determined by expert assessment and opinion surveys. It is constructed on the basis of a combination of 13 surveys and evaluations from 12 independent institutions specializing in governance and business environment analysis.

The Environmental Performance Index (EPI) is produced jointly by Yale University and Columbia University in collaboration with the World Economic Forum. It provides information at a national scale on how close countries are to achieving established environmental policy goals. It is divided into two pillars: environmental health and ecosystem vitality. In turn, these are divided into several categories. The present study uses the ecosystem vitality category, referring to the sustainability of agricultural resources for the considered 2012–2016 time period.

The values of these last three indices lie between 0 and 100, with a score of 100 corresponding to countries that are less corrupt whose food products register a high level of quality and safety, and whose agricultural resources score highly in terms of the environment. The main statistics for the criteria explained above are detailed in Table 5.

Given the particular features of each criterion and the objective of this research, the aim is to maximize the LPI, Q&S, CPI, and EPI, and minimize the number of notifications. In the years analyzed, the first three criteria to be maximized appear to remain fairly stable, whereas greater variations are observed in EPI and notifications. The latter two register a drastic reduction, as can be seen when comparing the maximum values. However, while the fall in the number of notifications represents an improvement in the food risk of imported products, the EPI indicates the opposite, i.e., it reflects the poorer environmental performance of the countries assessed. Nevertheless, this value may be due to methodological changes in the evaluation of agricultural issues introduced in the past year. The new indicator to capture the effects of nitrogen fertilizer, the sustainable nitrogen management index, replaces nitrogen use efficiency and nitrogen balance, which had been used up to that point. 

In 2012, the following countries achieved the highest scores on the criteria to be maximized: Singapore on the LPI and CPI; the USA on the Q&S; and Serbia, Argentina, and Singapore on the EPI. The aim of all exporters is for their products to reach destination countries in the best possible condition; as such, notifications are an obstacle that could hinder or even prevent the import of products. Many of the countries analyzed did not receive any notifications in 2012 (Russia, Canada, Switzerland, Serbia, Turkey, Kazakhstan, Cambodia, Mexico, Uruguay, Singapore, Chile, Egypt, Norway, Israel, and Indonesia), while others, such as Pakistan, India, and Thailand, had to deal with situations that seriously damaged their trade relations (8, 7, and 4 notifications, respectively).

Small variations are observed in 2014, with the USA and Switzerland leading the LPI, New Zealand the CPI, Cambodia the EPI, and the USA also heading up the Q&S. Regarding notifications, Serbia and Argentina receive the most. Lastly, in 2016, Singapore once again took the lead in logistics, Switzerland scored the highest in the CPI, the USA achieved the best score for the EPI and Q&S, and Argentina maintained its position as the country with the most notifications. However, in 2014 and 2016, around 60% of countries did not receive any notifications at all, and 36% received only one. In this respect, there is marked improvement in terms of a reduction in notifications for EU cereal imports.

## 4. Results and Discussion

The TOPSIS, ELECTRE, and CE models were applied to the countries presented in Table 3 (24, 22, and 25 countries for 2012, 2014, and 2016, respectively), which, together, comprise the different alternatives for the origin of EU cereal imports. The criteria that make it possible to distinguish between potential suppliers on the basis of their food risk are defined in Table 4. TOPSIS and ELECTRE require weights to be assigned to the criteria in order to rank their importance in the choice. It was decided to assign each of them the same relevance (0.2) so as not to distort the results compared to CE. On the other hand, CE needs the choice of inputs and outputs that define the hypothetical production function. The particular characteristics of each criterion led to the designation of past notifications as input, with the rest being output. It has been shown that in the construction of synthetic indices, this decision does not substantially modify the results [89].

The selected framework makes it possible to assess the risk associated with the institutional and economic conditions of the different suppliers and rank them accordingly. Overall, the results in Table 6 show that some countries are at the top/bottom of the ranking for all three years, regardless of the model used. For example, both the official trade statistics (following micro/macroeconomic criteria) and the TOPSIS, ELECTRE, and CE models place Canada and Switzerland among the most relevant suppliers of cereals to the EU, as well as the safest in terms of food risk. However, other countries such as the USA and Australia register greater variations with respect to the year of study and methodology. Furthermore, although Australia does not present a particular concern in terms of food risk, it does not rank very highly according to microeconomic criteria (11th, 17th, and 16th in Table 3); perhaps its remoteness, which ultimately entails higher trade costs, could be behind this result. On the other hand, trade with Ukraine, Serbia, Pakistan, Russia, and Thailand, shown by Comtrade to be the main suppliers of cereals to the EU, is not advisable according to the institutional food risk criteria.

However, analyzing the average results, the three models show that, along with Canada and Switzerland, the USA is one of the countries whose exports apparently present the lowest institutional risk; at the same time, it is one of the main suppliers of cereals to the EU. According to the multicriteria framework, trade with Pakistan, Cambodia, and Kazakhstan appears to be least advisable, yet their exports to the EU represent a considerable share of the total. In addition, if European importers gave greater weight to food risk-related issues, in no case would they choose Ukraine as the top supplier (Table 3); there would be a decrease in trade relations, with its position dropping to 18, 17, and 17, according to TOPSIS, ELECTRE, and CE, respectively.

MCDM methods have proven to be effective in prioritizing countries by taking into account structural drivers of food risk. The method could provide a picture that would match the actual trade ranking if only the selected criteria related to political and structural factors underlying food risk were considered in the importers’ decisions. Actually, Table 6 shows that countries occupying the top spots in the ranking are closely linked to the EU and have similar economic and social characteristics. Conversely, the bottom-ranked countries are economically less developed nations where food risk does not seem to be a priority (Pakistan, Vietnam, and Cambodia, among others).

However, the actual ranking of EU cereal suppliers depends on other considerations, in particular, trade costs. This is illustrated by the different positions that cereal suppliers occupy according to the trade agreements signed with the EU. Thus, the results highlight differing situations, such as those of Switzerland and Norway, which are members of the European Free Trade Association (EFTA), but which occupy very distinct positions as cereal suppliers. Table 3 shows that Switzerland ranks fifth in terms of volume of EU cereal imports; however, according to food risk criteria and the three MCDM methods, its position rises to second with TOPSIS and third with ELECTRE and CE. On the other hand, Norway, although it ranks among the top 10 suppliers in terms of food risk (eighth in TOPSIS, fourth in ELECTRE, and seventh in CE), is not currently one of the EU’s main suppliers (22nd place in 2014 and 2016; Table 3). Neither Norway nor Switzerland received any notifications during the three years analyzed. Moreover, they have demonstrated high levels of logistics development, low levels of corruption, and high levels of perceived food quality, and their agriculture scores well on the environmental sustainability index.

Another country that merits attention is Singapore, which is the EU’s most important trading partner among all the ASEAN members. This successful relationship led to the signing of an FTA in late 2019, which represents a step forward in their bilateral trade, opening up the possibility of eliminating customs duties on imports, as well as other measures to boost imports of goods (Council Decision (EU) 2019/1875 of 8 November 2019). This Asian country did not receive any notifications in the three years analyzed. It also ranked first on the LPI, scored highly on the Q&S 2016, and registered the best corruption score in 2012. However, its cereal exports to the EU were around just 0.6% of the total EU’s cereal imports in 2016, and even lower in 2014 and 2012 (0.2% and 0.4%, respectively). Given the less relevant position of Singapore as a cereal producer, it is not expected that the FTA and the country’s favorable position in the risk safety ranking will significantly boost its exports to the EU.

**Figure 3:**
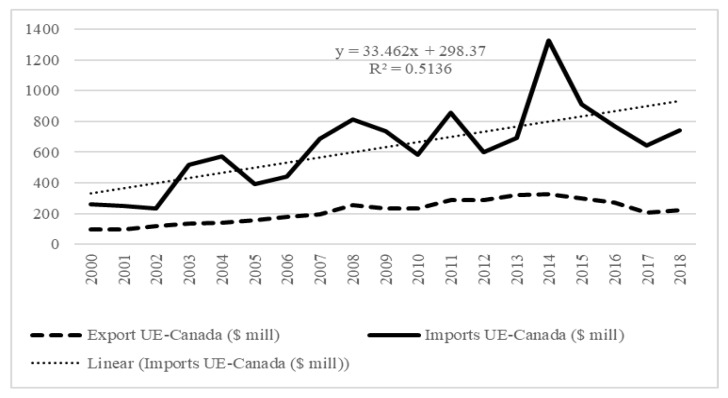
Evolution of bilateral trade in cereals between the EU and Canada. Source: Authors’ elaboration.

Furthermore, bilateral relations between the EU and Canada are currently bolstered by the Comprehensive Economic and Trade Agreement (CETA). Under this agreement, import quotas and tariff rates will be progressively reduced and eventually eliminated entirely [90]. Figure 3 shows a positive trend in EU imports from Canada.

The nearly 20-year period analyzed demonstrates the EU’s trade deficit with respect to the Canadian market, indicating the importance of Canada as an international supplier of cereals. The trend equation confirms the progressive increase over time, a trend which will be substantially bolstered by the aforementioned trade agreements. In this case, the favorable risk assessment supports the hypothesis that Canada will improve its competitive position in the EU market.

A country’s income level can be related to some extent to domestic capacity and infrastructure for food and feed quality and control. Therefore, in line with the objectives of this paper, ANOVA was carried out to test the null hypothesis of independence between countries’ positions in the ranking and their income levels. To that end, the mean CE values for three years were used, along with the World Bank’s country classifications by income level for 2016 (Table 7).

Analysis of variance (ANOVA) divides the variation in the ranking into two categories: between-group variation (if it is due to differences between groups) and within-group variation (if it is due to differences within each group). As shown in column 6 of Table 8, the results confirm the alternative hypothesis; it can thus be concluded that a country’s income level affects the position it achieves in the ranking.

The Tukey–Kramer test was then used to determine which groups differ from each other and their quantification in terms of CE, which would mean that they would occupy different positions in the ranking.

The results reveal the existence of two clearly differentiated subsets: one consisting of countries classified as lower-middle income, and the other consisting of upper-middle and high income. In addition, the EC score rises from 0.6661 to 0.8468, corresponding to the income groups at the extremes (Table 9). In the bottom part of the table, these differences are quantified by confidence intervals. Thus, for example, the average difference between high and lower-middle income is 0.1907, with a confidence interval of [0.0957, 0.2856], i.e., the minimum difference is 0.0957 and the maximum difference is 0.2856, at a confidence level of 95%.

In summary, countries with better capacity to manage food safety will normally be more qualified to achieve better positions in multicriteria food risk assessment. MCDM can easily be applied by panels of experts from cereal importing firms. The approach offers a structured method with a prevention tool that considers country risks and can modify selections based exclusively on market variables such as price and volume. Validation of the framework for cereals suggests that scaling up the method to other value chains would be straightforward.

As suggested, actual purchasing choices consider other variables not related to supplier risk. An implication of the study is that actual trade choices would not exactly match the results of the risk evaluation. However, actual import decisions must be weighed with the results of the risk evaluation assessments, which can become a crucial component of trading decisions. Although up to the present, supplier costs have been a determining factor of import choices, in future, food safety considerations could have increased influence, which means further difficulties for low-income countries.

## 5. Conclusions

The globalization of markets and advances in logistics have meant that distance is no longer a barrier to international trade; however, this has inherent problems, such as those associated with the entry of products which are in poor condition. As health reaches paramount importance in future trade relations, food risk is a factor that should be prioritized in the process of choosing a supplier country for a particular food, since a population’s health depends on the quality of the food it eats. This paper suggests an MCDM methodology that can be easily extended to risk prevention strategies for food traders. Our methodology supplies a tool that takes into account several markers of food safety risk related to the exporting country’s conditions. 

This research proposes the use of three MCDM methods to establish a ranking of countries that supply cereals to the EU, applying choice criteria based on food risk assessment. The results reveal clear differences between the classification carried out according to purely micro/macroeconomic patterns compared to the proposed criteria. Thus, for example, Ukraine, the main exporter of cereals, would not be in the top position according to the food risk criteria (occupying position 18 in the ranking according to TOPSIS and 17 according to ELECTRE and TOPSIS), while Norway would move upward in the ranking (position 4 according to ELECTRE), even though it does not currently supply large volumes of cereal to the EU (position 22 in the supplier ranking in 2012 and 2016).

Although the rankings obtained using the different methods are not the same, they do show certain similarities, revealing the most suitable countries according to their level of food risk. All the countries in the top positions have been described by the World Bank as high-income. They are developed economies whose distance from the EU does not present an obstacle. However, Asian and South American countries should seek improvements in all food risk criteria in order to strengthen trade relations with Europe. Beyond the usefulness of the proposed methodological framework, a possible policy implication of the analysis is that, in the future, strengthening food safety considerations in importer choices could involve further difficulties for lower-income exporters.

This contribution supplies a framework for institutional risk assessments of food safety that can be further developed with more focused indicators from international databases. The approach offers a structured method with a tool for supplier selection that considers country risks, and could be extended to a wide range of food value chains.

The research carried out is not without its limitations; the use of synthetic indices to assess certain aspects of the countries, such as logistics, quality, and sustainability, could be criticized, and these could be adapted to the specific conditions of the studied value chains. These indicators are strongly conditioned, not only by possible errors in the sample, but also by the treatment of the variables and their weights, leading to a loss of rigor in clarifying the characteristics of each country. Risk factors can also be influenced by local shocks to the economy or the sanitary conditions of exporting countries, such as crop shortage or plant disease. We believe that the MCDM framework can be adapted to these circumstances and extended to different value chains. Finally, in future research, a more specific analysis should be carried out, focusing on certain geographic areas where the availability of official statistics would give rise to a more in-depth study.

## Figures and Tables

**Table 1:** Overview of papers on decision processes in the agricultural and food sectors.

AUTHORS	RESEARCH OBJECTIVES	METHODOLOGY	CONCLUSIONS
Mavi et al. (2016) [54]	Supplier selection in supply chain risk management	Shannon entropy Fuzzy TOPSIS	Demand risk is the most important factor
Montgomery et al. (2016) [55]	Evaluate agricultural land capability and suitability	GIS-Logic Scoring of Preference	The model is an effective tool for integrated regional land-use planning
Debnath et al. (2017) [56]	Recognize and select the valuation criteria for strategic project portfolio selection of agro byproducts	Grey DEMATEL-MABAC	The genetically modified agro by-products are found to be the best portfolio.
Seyedmohammadi et al. (2018) [57]	Evaluate areas suitable for cultivation priority planning	SAW, TOPSIS, Fuzzy TOPSIS	Fuzzy TOPSIS results were more accurate than the others
Rostamzadeh et al. (2018) [58]	Develop a framework for the sustainable supply chain risk management evaluation.	FTOPSIS-CRITIC	The most important criteria are sustainable production/manufacturer risks, while sustainable recycling risk is the least important one
Raut et al. (2018) [59]	Identify the factors of postharvest losses in the fruits and vegetables supply chain	AHP	(1) Lack of linkages between institution, industry, and government, (2) climate and weather conditions, (3) lack of linkages in the marketing channel are the three top factors.
Qureshi et al. (2018) [60]	Focuses on the crop selection pattern in Indian environment	Fuzzy TOPSIS	The scarce availability of resources to Indian farmers poses many challenges to farming practices which most need sustainability
Rao et al. (2019) [61]	Identify indicators for development of climate resilient agriculture	WSM, AHP	Identifies a list of 30 sustainability indicators for climate resilient agriculture
Paul et al. (2020) [62]	Evaluate the potentiality of reclaimed water use for agricultural irrigation	AHP	Spatial distribution of suitable areas for water reuse is closely linked to the agricultural areas
Garcia-Alvarez-Coque et al. (2020) [27]	Evaluate social, health and environmental criteria for dietary patterns	AHP	Mediterranean diet adapts well to urban multiactor priorities.
Balezentis et al. (2020) [63]	Assessment of crop farming sustainability	SAW, TOPSIS, EDAS	Scenarios minimizing labor use yield the most sustainable crop-mix

Source: Authors’ elaboration.

**Table 2:** Comparison of multicriteria decision-making methods.

Method	Description	Advantages	Disadvantages
ELECTRE (Roy, 1973, 1991) [79,80]	Uses outranking classification method, pairwise comparison, and compensatory method	- Can be applied even when there is information missing. - Compares alternatives that are not directly comparable - Used for quantitative and qualitative attributes	- Time-consuming without the use of software
TOPSIS (Yoon and Hwang, 1985) [81]	Assessment based on the compensatory method; Measures the distance of the alternatives from the ideal solution	- A bad result on one criterion offsets a good one on another criterion - Accounts for positive and negative ideal solutions	- Requires normalization in multidimensional problems
Cross-efficiency (Sexton et al., 1986; Doyle and Green, 1994) [82,83]	Provides a peer evaluation such that each unit is assessed with respect to the weights of the other units in the sample.	- Creates a complete ranking of all observations. - Does not require the alternatives to be weighted	- Requires homogeneity

Source: Authors’ elaboration.

**Table 3:** Ranking of suppliers of cereals to the EU according to their volume exported.

Rank Order	2012	2014	2016
1	Ukraine	Ukraine	Ukraine
2	Russian Fed.	Canada	Canada
3	USA	USA	USA
4	Canada	Russian Fed.	Russian Fed.
5	Switzerland	Switzerland	Switzerland
6	Serbia	Serbia	Thailand
7	Thailand	Pakistan	Brazil
8	Argentina	Turkey	Turkey
9	Brazil	Brazil	Cambodia
10	Turkey	Cambodia	Serbia
11	Australia	Argentina	Pakistan
12	Kazakhstan	Chile	Argentina
13	India	Myanmar	Mexico
14	Cambodia	Peru	Myanmar
15	Pakistan	Mexico	Peru
16	Mexico	South Africa	Australia
17	Uruguay	Australia	Viet Nam
18	Viet Nam	Bolivia	Kazakhstan
19	Singapore	New Zealand	Singapore
20	Chile	Uruguay	Uruguay
21	Egypt	Egypt	Chile
22	Norway	Indonesia	Norway
23	Israel		Egypt
24	Indonesia		Israel
25			Bolivia

Source: Authors’ elaboration. Eurostat data.

**Table 4:** Definitions of criteria.

Criterion	Source	Unit Measured
Notifications	RASFF	No. Notifications
Logistics Performance Index (LPI)	World Bank	Score of 1–5
Quality & Safety Index (Q&S)	The Economist Intelligence Unit	Scale from 0 to 100
Corruption Perceptions Index (CPI)	Transparency International.	Scale from 0 to 100
Environmental Performance Index (EPI) Agriculture	Yale Centre for Env. Law and Policy	Scale from 0 to 100

Source: Authors’ elaboration.

**Table 5:** Main statistics for criteria in sample of EU cereal suppliers.

*Statistic*	2012				
	Notifications	LPI	Q&S	CPI	EPI
Mean	1.33	3.24	65.93	50.13	61.19
Max	8.00	4.00	88.10	87.00	96.00
Min	0.00	2.68	26.80	22.00	14.66
St. Dev.	2.28	0.44	16.37	23.55	28.28
	**2014**				
	**Notifications**	**LPI**	**Q&S**	**CPI**	**EPI**
Mean	0.36	3.15	65.56	48.18	83.57
Max	2.00	3.99	87.00	90.00	100.00
Min	0.00	2.25	28.00	21.00	41.21
St. Dev.	0.66	0.50	15.35	22.14	18.33
	**2016**				
	**Notifications**	**LPI**	**Q&S**	**CPI**	**EPI**
Mean	0.44	3.10	67.38	48.72	43.14
Max	2.00	4.00	86.70	86.00	72.38
Min	0.00	2.30	34.70	21.00	4.59
St. Dev.	0.58	0.51	14.11	22.42	16.74

Source: Authors’ elaboration from data sources in Table 4.

**Table 6:** Ranking of cereal suppliers to the EU-28.

TOPSIS			ELECTRE			CE			Mean		
2012	2014	2016	2012	2014	2016	2012	2014	2016	TOPSIS	ELECTRE	CE
Singapore	Switzerland	USA	Singapore	Canada	Canada	Singapore	USA	USA	Canada	Canada	USA
Canada	Canada	Canada	Canada	USA	USA	USA	Argentina	Canada	Switzerland	USA	Canada
Chile	Australia	Uruguay	Switzerland	Switzerland	Switzerland	Canada	Canada	Argentina	N. Zealand	Switzerland	Switzerland
Australia	USA	Switzerland	Norway	Australia	Australia	Switzerland	Switzerland	Switzerland	Australia	Norway	Argentina
Switzerland	N. Zealand	Australia	USA	N. Zealand	Norway	Thailand	Australia	Australia	USA	N Zealand	Singapore
Uruguay	Uruguay	Norway	Chile	Uruguay	Uruguay	Norway	Serbia	Norway	Uruguay	Australia	S. Africa
Norway	Chile	Chile	Turkey	Russian F	Chile	Turkey	S. Africa	Brazil	Singapore	Singapore	Norway
Serbia	Brazil	Israel	Thailand	Mexico	Israel	Chile	Turkey	Singapore	Norway	Uruguay	Australia
Turkey	Indonesia	Ukraine	Australia	S. Africa	Ukraine	India	Uruguay	Turkey	Chile	Chile	Turkey
Egypt	Peru	Egypt	Uruguay	Brazil	Brazil	Argentina	Ukraine	Israel	Israel	S. Africa	N Zealand
Mexico	Mexico	Bolivia	Mexico	Turkey	Singapore	Uruguay	N. Zealand	Russian F	Egypt	Mexico	Thailand
Israel	Russian F	Singapore	Egypt	Indonesia	Mexico	Mexico	Russian F	Chile	Mexico	Turkey	Uruguay
Indonesia	Bolivia	Myanmar	Serbia	Cambodia	Egypt	Brazil	Mexico	Uruguay	Indonesia	Brazil	India
Brazil	Egypt	Mexico	Argentina	Chile	Turkey	Australia	Brazil	Serbia	Bolivia	Israel	Serbia
Ukraine	Myanmar	Kazakhstan	Brazil	Peru	Serbia	Israel	Bolivia	Viet Nam	Myanmar	Russian F	Brazil
Kazakhstan	Cambodia	Brazil	Israel	Myanmar	Argentina	Egypt	Myanmar	Thailand	Kazakhstan	Thailand	Chile
Cambodia	S. Africa	Serbia	Ukraine	Argentina	Bolivia	Ukraine	Peru	Mexico	Brazil	Ukraine	Ukraine
USA	Turkey	Turkey	Indonesia	Ukraine	Russian F	Pakistan	Indonesia	Ukraine	Ukraine	Egypt	Israel
Russian F	Ukraine	Russian F	India	Bolivia	Thailand	Serbia	Cambodia	Peru	Russian F	Indonesia	Mexico
Argentina	Pakistan	Viet Nam	Kazakhstan	Egypt	Viet Nam	Viet Nam	Chile	Egypt	Turkey	Argentina	Russian F
Viet Nam	Argentina	Cambodia	Viet Nam	Serbia	Myanmar	Russian F	Egypt	Bolivia	Cambodia	Serbia	Peru
Thailand	Serbia	Thailand	Russian F	Pakistan	Kazakhstan	Kazakhstan	Pakistan	Myanmar	Peru	Bolivia	Viet Nam
India		Peru	Cambodia		Peru	Indonesia		Kazakhstan	Viet Nam	Myanmar	Bolivia
Pakistan		Pakistan	Pakistan		Cambodia	Cambodia		Cambodia	S. Africa	Peru	Myanmar
		Argentina			Pakistan			Pakistan	Serbia	India	Egypt
									Thailand	Cambodia	Indonesia
									Argentina	Viet Nam	Pakistan
									Pakistan	Kazakhstan	Cambodia
									India	Pakistan	Kazakhstan

Source: Authors’ elaboration.

**Table 7:** Classification of countries by income level.

High Income	Lower-Middle Income	Upper-Middle Income
Singapore	Viet Nam	Thailand
Australia	Indonesia	Serbia
New Zealand	Myanmar	Turkey
Switzerland	Cambodia	Russian Fed
Norway	Ukraine	Kazakhstan
Uruguay	Bolivia	Mexico
Chile	Egypt	Argentina
Israel	India	Brazil
USA	Pakistan	Peru
Canada		South Africa

Source: Authors’ elaboration. World Bank data.

**Table 8:** Analysis of variance: CE ranking by income level.

Type of Differences	Df	SumSq	MeanSq	F-Value	*p*-Value	
inter-group	2	0.172	0.086	12.453	0.000	***
intra-group	26	0.180	0.007			
Total	28	0.352				

*** The differences inter-group is significant at the 0.01 level; Source: Authors’ elaboration.

**Table 9:** Differences in ranking by income.

Country Group	N	1	2	
Lower-middle income	9	0.6661		
Upper-middle income	10		0.7583	
High income	10		0.8468	
**Comparison (income)**		**Difference**	**Lower**	**Upper**
High income	Lower-middle income Upper-middle income	0.1907 * 0.0885	0.0957 −0.0039	0.2856 0.1809
Lower-middle income	High income Upper-middle income	−0.1907 * −0.1022 *	−0.2856 −0.1971	−0.0957 −0.0072
Upper-middle income	High income Lower-middle income	−0.0885 0.1021 *	−0.1809 0.0072	0.0039 0.1971

* The mean difference is significant at the 0.05 level; Source: Authors’ elaboration.

## Appendix A

### Appendix A.1. TOPSIS Method

The Technique for Order Preference by Similarity to the Ideal Solution (TOPSIS) was first proposed by [91]. This method is based on the concept that the chosen alternative should have the shortest geometric distance from the positive ideal solution and the longest geometric distance from the negative ideal solution. An assumption of TOPSIS is that the criteria are monotonically increasing or decreasing.

TOPSIS has two main advantages: its mathematical simplicity and substantial flexibility in the definition of the choice set. The TOPSIS method consists of six consecutive stages [92]:

- Calculate the normalized decision matrix.
- Calculate the weighted normalized decision matrix.
- Calculate ideal and negative ideal solutions.
- Calculate the separation measures.
- Calculate the relative closeness to the ideal solution.
- Rank the preference order.

The preference order of the alternatives, in accordance with their relative closeness to the ideal solution, is obtained. A higher value of relative closeness indicates a higher preference order among generated design alternatives, meaning it is preferred [93,94].

### Appendix A.2. ELECTRE Method

*Elimination et Choix Traduisant la Realité* (ELECTRE) was proposed by [95]. ELECTRE is an outranking method that discards unacceptable alternatives and uses another multicriteria decision-making method to select the best one. The limited set of alternatives obtained saves a lot of time in selection [96]. This method establishes a preference relation between a set of solutions where each one shows a degree of dominance over the others with respect to a criterion [97].

- Implementing this method involves the following steps:
- Calculate the decision matrix.
- Calculate the normalized decision matrix using the ratio of the range.
- Calculate the weighted normalized decision matrix
- Calculate the concordance index matrix.
- Calculate the discordance index matrix.
- Set the threshold values: a minimum for concordance and a maximum for discordance.
- Calculate the concordance dominance matrix
- Calculate the discordance dominance matrix.
- Calculate the aggregate dominance matrix
- Interpret the values of the aggregate dominance matrix.

### Appendix A.3. DEA Model: Cross-Efficiency

DEA is a nonparametric mathematical programming system used to evaluate a set of comparable decision-making units (DMUs). It is used to calculate the maximum performance (output) given a certain amount of input associated with each DMU; or vice versa, the minimum input needed to achieve the established output. [98] was the first to measure technical efficiency, in 1957, with [99] subsequently developing the CCR model under the assumption that production exhibits constant returns to scale. As an extension, [100] applied the BCC model to account for the possibility that production has variable returns to scale. DEA differentiates between efficient and inefficient observations, but it cannot establish a ranking of the former. Thus, this study uses an extension of DEA, namely cross-efficiency (CE), in order to obtain a complete ranking of all DMUs according to the inputs/outputs that define them.

CE was originally proposed by [82], and later validated by [83], as a way to overcome the main limitations of DEA. As highlighted by [101], these limitations include not only the inability to distinguish between efficient units, but also the fact that an inappropriate weighting scheme can distort the results. CE is used to assess the performance of each country, computed using the optimal input and output weights for the other countries. The resulting CE matrix contains information on the efficiency of a country relative to the others. This allows the researcher to rank all the observations that have an efficiency score of 1. Each element is calculated by means of the following expression:

(1) $$E_{kj}=\frac{\sum_{r=1}^{s}u_{rk}y_{rj}}{\sum_{i=1}^{m}v_{ik}x_{ij}}$$

*j = 1…,n; k =1,…,n* (2)

where *u_rk_* *y* *v_ik_* are the optimal multipliers obtained by DEA for the corresponding country, with the original efficiency scores on the diagonal. Thus, the value of *E_kj_* is obtained by evaluating country *j* using the optimum weights for country *k* (DEAR software [102] was used to calculate the efficiency level of each country analyzed).

Since the objective is not to measure efficiency, but rather to establish a ranking of alternatives, the determination of variables corresponding to the inputs/outputs is an arbitrary decision made by the researcher and does not substantially affect the conclusions drawn [103,104]. The criteria used as inputs are transformed into “values to be improved” by subtracting from the maximum value of each criterion the value corresponding to each DMU [89]. The use of these three methods will yield more complete results, providing good information for decision-making.
